# *N*-Terminal Protease Gene Phylogeny Reveals the Potential for Novel Cyanobactin Diversity in Cyanobacteria

**DOI:** 10.3390/md11124902

**Published:** 2013-12-09

**Authors:** Joana Martins, Pedro N. Leão, Vitor Ramos, Vitor Vasconcelos

**Affiliations:** 1 Faculty of Sciences, University of Porto, Rua do Campo Alegre, Porto 4169-007, Portugal; E-Mails: joana.martins@ciimar.up.pt (J.M.); vtr.rms@gmail.com (V.R.); 2 CIIMAR/CIMAR—Interdisciplinary Centre of Marine and Environmental Research, University of Porto, Rua dos Bragas 289, Porto 4050-123, Portugal; E-Mail: pleao@ciimar.up.pt

**Keywords:** cyanobacteria, cyanobactins, bioactive compounds, phylogeny

## Abstract

Cyanobactins are a recently recognized group of ribosomal cyclic peptides produced by cyanobacteria, which have been studied because of their interesting biological activities. Here, we have used a PCR-based approach to detect the *N*-terminal protease (A) gene from cyanobactin synthetase gene clusters, in a set of diverse cyanobacteria from our culture collection (Laboratory of Ecotoxicology, Genomics and Evolution (LEGE) CC). Homologues of this gene were found in *Microcystis* and *Rivularia* strains, and for the first time in *Cuspidothrix*, *Phormidium* and *Sphaerospermopsis* strains. Phylogenetic relationships inferred from available A-gene sequences, including those obtained in this work, revealed two new groups of phylotypes, harboring *Phormidium*, *Sphaerospermopsis* and *Rivularia* LEGE isolates. Thus, this study shows that, using underexplored cyanobacterial strains, it is still possible to expand the known genetic diversity of genes involved in cyanobactin biosynthesis.

## 1. Introduction

Cyanobacteria are one of the preferred microbial sources in the search of novel bioactive compounds due to the great structural diversity of their secondary metabolites [1,2]. Molecules derived from polyketide synthase (PKS), non-ribosomal peptide synthetase (NRPS) and hybrid PKS/NRPS biosynthetic pathways constitute the majority of known cyanobacterial natural products [2].

Recently, several studies have focused on an emerging group of low-molecular weight cyclic peptides of ribosomal origin. These are structurally and biosynthetically related, thus justifying the establishment of a new metabolite family—the cyanobactins [3,4,5]. While many cyanobactins are simple peptide macrocycles (*N*-*C* terminal cyclized), most are additionally decorated with diverse posttranslational modifications, such as heterocyclization, isoprenylation or *N-*methylation, making this group of compounds one of the largest classes of diverse peptides [4,6]. Several pharmacological relevant activities, including cytotoxicity [7], antimalarial [8,9] and multidrug reversing activities [10] have been reported for cyanobactins. As an example, trunkamide, produced by *Prochloron* sp., was shown to be highly selective for the human renal UO-31 cell line and to have a favorable COMPARE profile at the National Cancer Institute, being listed as a pre-clinical candidate [11]. Recent studies, using a large diversity spectrum, have estimated that these peptides are found in 10%–30% of all cyanobacteria [1,6], being widespread among symbiotic as well as free-living cyanobacteria isolated from terrestrial, freshwater and marine environments [1,4,12].

Cyanobactin biosynthetic gene clusters, and their respective metabolites, have been described in cyanobacteria belonging to the unicellular genera *Prochloron* (patellamide, lissoclinamides, ulithiacyclamides, patellin and trunkamide) [13], *Microcystis* (microcyclamide, piricyclamide) [13,14] and *Cyanothece* (cyanothecamides) [6,15]; the filamentous non-heterocystous genera *Trichodesmium* (trichamide) [13], *Planktothrix* (prenylagaramide) [6,16], *Lyngbya* (aesturamide) [3,17], *Oscillatoria* (a full gene cluster was discovered, however the corresponding product has not been found) [6] and *Arthrospira* (arthrospiramide) [6]; and the filamentous heterocystous *Anabaena* (anacyclamide) [13] and *Nostoc* (tenuecyclamide) [13]. The patellamide gene cluster (*pat*), from the ascidian symbionts *Prochloron* spp., was the first cyanobactin biosynthetic gene cluster to be described [18]. Most of the homologous clusters reported since then resemble *pat*, although the gene arrangement is not precisely conserved [6,13]. Nevertheless, all clusters feature genes coding for two proteases (*N*-terminal protease—A, and *C*-terminal protease—G), which cleave precursor peptides (E) and macrocyclize the resulting fragments. Ribosomally translated precursor peptides, act as substrates for the posttranslational modifications, which imprint structural diversity to these metabolites. Furthermore, cyanobactin precursor peptides are hypervariable, meaning that identical enzymes can often synthesize numerous products depending on discrete changes in small cassettes [19]. The minimal set of genes present in cyanobactin gene clusters seems to include, apart from genes encoding A, G and E, two genes coding for short conserved hypothetical proteins (B and C) of unknown function [6]. Additionally, the cyclodehydratase (D) gene, responsible for heterocyclization and the prenyltransferase (F) gene, responsible for prenylation, may be present in the gene cluster, as well as thiazoline/oxazoline dehydrogenases, methyltransferases and other non-characterized proteins [20]. More recently, cyanobactins with more than one precursor peptide have been described [6].

Evidence from genomic studies [6,13] points towards the existence of a large number of cyanobactins yet to be discovered. With this in mind, we have employed a PCR-based strategy to identify potential cyanobactin producing strains in our culture collection. To this end, we designed degenerate oligonucleotide primers that were used to detect gene A from cyanobactin gene clusters among a set of diverse cyanobacteria. Phylogenetic relationships were inferred using all the available A-gene sequences (including sequences from this study), which ultimately led to the identification of promising cyanobacterial strains/phylotypes from which novel cyanobactin chemotypes may be discovered.

## 2. Results

### 2.1. PCR Analyses

The selected cyanobacterial strains (see  Experimental Section) were screened by PCR for the presence of cyanobactin *N*-terminal protease (A) gene. Apart from the strain used as positive control (*M. aeruginosa* LEGE 91351), this gene was detected in 11 other strains (Table 1). We were unable to detect gene A in cyanobacteria belonging to the order Pleurocapsales. A-gene sequences were obtained for strains belonging to the unicellular genus *Microcystis* (six isolates, including positive control, order Chroococcales), the filamentous non-heterocystous genus *Phormidium* (three isolates, order Oscillatoriales), and one isolate of each of the nostocalean genera *Cuspidothrix*, *Sphaerospermopsis* and *Rivularia*. The majority of these isolates were obtained from freshwater environments, with the exception of the marine strain *Rivularia* sp. LEGE 07159 (Table 1).

**Table 1:** Cyanobacterial strains positive for cyanobactin *N*-terminal protease (A) gene.

Strain	Habitat ^a^	Source ^b^	Accession Number	
			16S rRNA	(A) gene
*Cuspidothrix* sp. LEGE 03284	F	Montargil, Portugal	KC989703	KF008260
*Microcystis aeruginosa* LEGE 91347	F	Reservoir, Bemposta dam, Portugal	KC989705	KF008262
*Microcystis aeruginosa* LEGE 91351	F	Pond, Lagoa das Braças, Portugal	KC311966	KF008265
*Microcystis aeruginosa* LMECYA 1	F	Reservoir, Montargil dam, Portugal	KC989706	KF008263
*Microcystis* sp. IZANCYA 45	F	Pond, Lagoa da Vela, Portugal	KC311968	KF008266
*Microcystis* sp. LEGE 08328	F	Lake Zumpango, Mexico	KC989704	KF008261
*Microcystis* sp. LEGE 08331	F	Man-made channel, Cuemanco, Mexico	KC989707	KF008264
*Phormidium* sp. LEGE 06204	F	WWTP, Febros river, Portugal	KC989699	KF008256
*Phormidium* sp. LEGE 06363	F	WWTP, Febros river, Portugal	KC989700	KF008257
*Phormidium* sp. LEGE 07215	F	WWTP, Febros river, Portugal	KC989698	KF008255
*Rivularia* sp. LEGE 07159	M	Beach, Burgau, Portugal	KC989702	KF008259
*Sphaerospermopsis* sp. LEGE 00249	F	Reservoir, Maranhão dam, Portugal	KC989701	KF008258

**^a^** M—marine, F—freshwater; **^b^** WWTP—Wastewater Treatment Plant.

### 2.2. Phylogenetic Analyses

**Figure 1:**
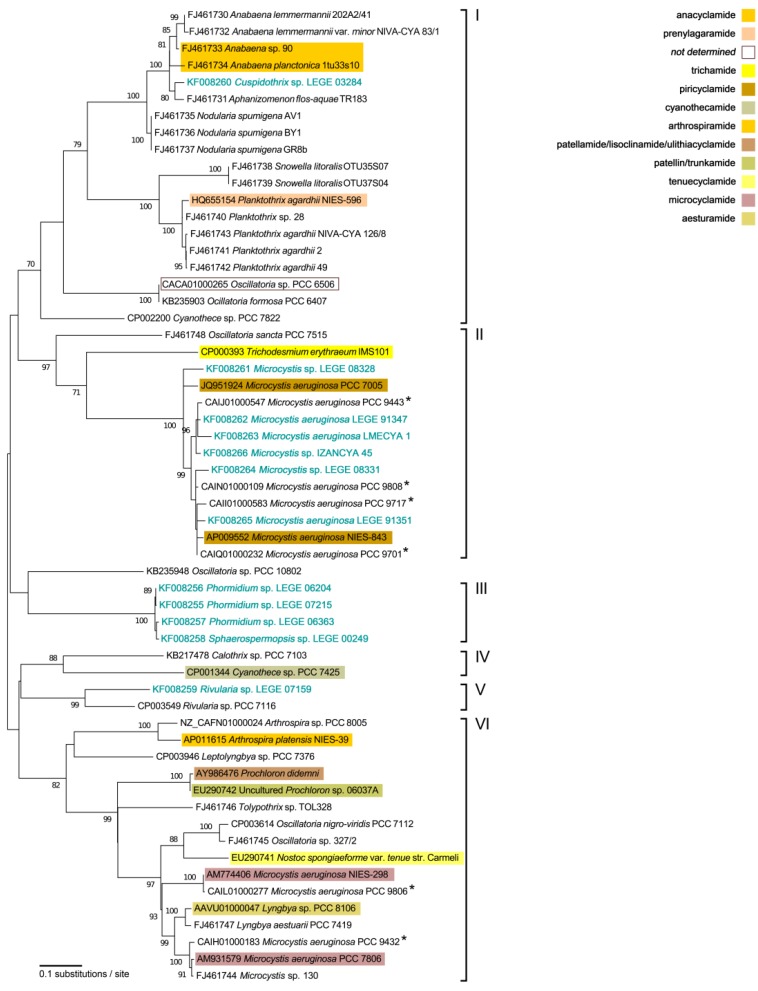
Unrooted maximum-likelihood phylogenetic tree of partial cyanobactin *N*-terminal protease (A) gene sequences (819 bp). The six clusters are identified by I–VI. Numbers along branches indicate the percentage of bootstrap support considering 1000 pseudo-replicates: only those equal to or higher than 70% are indicated. Strains from this study are indicated in light blue, whereas strains producing known cyanobactins are shaded with different colors. Strain *Oscillatoria* sp. PCC 6506 contains the cyanobactin *osc* gene cluster but its product remains unknown (see text for details). Cyanobactin gene cluster from strain *Microcystis aeruginosa* NIES-843 is inactive [14]. Strains marked with an asterisk correspond to isolates with A-genes identified through antiSmash searches, not previously described in the literature.

The phylogeny for gene A (Figure 1), constructed from 58 cyanobacterial strains sequences (12 from this study) revealed six distinct clades, three of them (I, II and VI) encompassing a large number of sequences. Clade I (ML bootstrap support value of 70%) is formed by the unicellular *Cyanothece* sp. PCC 7822, one subclade of filamentous non-heterocystous *Oscillatoria* spp. (one of these—strain PCC 6506—known to contain the cyanobactin *osc* gene cluster) and a larger group branched in two major subclades, which in turn form two smaller groups each. One of the branches is composed only by filamentous heterocystous cyanobacteria. It contains a subclade with *Cuspidothrix* sp. LEGE 03284 and isolates assigned to *Anabaena* and *Aphanizomenon* spp., which include the two strains known to produce anacyclamides (*Anabaena* sp. 90 and *Anabaena planctonica* 1tu33s10). In addition, it includes a subclade of three *Nodularia spumigena* strains. The other branch encompasses a subclade with two unicellular *Snowella litoralis* isolates, and also a subclade formed by the filamentous non-heterocystous genus *Planktothrix*, including the prenylagaramide-producing strain NIES-596. Clade II is formed by a broad subclade of unicellular *Microcystis* spp. sequences and by two phylogenetically distant sequences from distinct oscillatorialean cyanobacteria (including the trichamide-producing strain *Trichodesmium erythraeum* IMS101). The subclade of cyanobactin A-gene sequences from *Microcystis* spp. is one of the two clusters comprising members of this genus featured in the phylogenetic tree. This particular cluster (in Clade II) includes the six *Microcystis* strains from this study and also two *M. aeruginosa* strains known to contain the piricyclamide (*pir*) gene cluster. Clade III comprises the sequences of the three filamentous non-heterocystous *Phormidium* spp. LEGE CC strains and that from the filamentous heterocystous *Sphaerospermopsis* sp. LEGE 00249. The unicellular, cyanothecamide-producing *Cyanothece* sp. PCC 7425 grouped with the filamentous heterocystous *Calothrix* sp. PCC 7103 (although with a large genetic distance), forming Clade IV. Clade V is also constituted by two sequences. However, in this case the genetic distance is less pronounced and the strains, *Rivularia* sp. LEGE 07159 and PCC 7116 belong to the same genus. Clade VI constitutes the most heterogeneous cluster in terms of cyanobacterial and cyanobactin diversities. As observed in Clade IV, this clade does not comprise any of the strains screened in this study. It includes the ungrouped sequence from *Leptolyngbya* sp. PCC 7376, a subclade with two strains from the likewise oscillatorialean genus *Arthrospira*, including the arthrospiramide producer *A. platensis* NIES-39, and a larger subclade. This latter subclade harbors the ungrouped sequence from the nostocalean strain *Tolypothrix* sp. TOL328, a subclade with two *Prochloron* spp., which are unicellular marine cyanobacteria living symbiotically with ascidians and notably produce several different cyanobactins, and a still broad subclade divided in two main branches. The first forms a subclade with the sequences from other two strains of *Oscillatoria* spp. and from a filamentous heterocystous *Nostoc* species. This nostocalean strain is known to produce the tenuecyclamides. The second branch includes the remaining sequences from *Microcystis* strains. In this case, the isolates belonging to this genus are placed separately in two small clusters, each of them with a strain known to produce microcyclamide cyanobactins. These two groups of sequences are interleaved with those of two *Lyngbya* spp. (Oscillatoriales), one of which (strain PCC 8106) produces aesturamide. Finally, *Oscillatoria* sp. PCC 10802 is placed as a “loner sequence” (*i.e.*, having no close relatives) in the cyanobactin gene A phylogeny.

**Figure 2:**
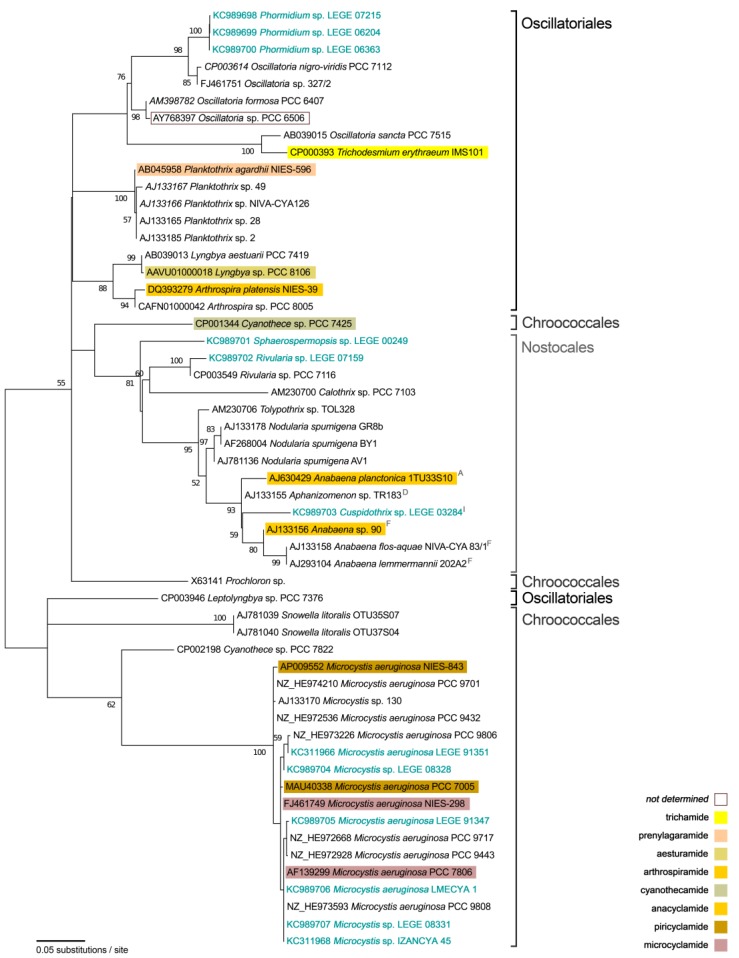
Unrooted maximum-likelihood phylogenetic tree of partial 16S rRNA gene sequences (760 bp). Numbers along branches indicate the percentage of bootstrap support considering 1000 pseudo-replicates: only those equal to or higher than 50% are indicated. Strains from this study are indicated in light blue. A, D, F and I refer to clades identified by Rajaniemni *et al*. [21]. Note: 16S rRNA gene sequences of *Oscillatoria* sp. PCC 10802, *Nostoc spongiaeforme* var. *tenue* str. Carmeli, *Prochloron didemni* and *Prochloron* sp. 06037A are not available and for that reason were not included in the tree.

On the other hand, the phylogenetic tree of 16S rRNA gene sequences (Figure 2) revealed a clearly different topology. It covers almost all the strains included in the A-gene phylogeny (Figure 1), with the exception of four cyanobacteria for which the sequences were not available. In this case, all the 17 *Microcystis* strains show to be phylogenetically very close, clustering in a tight and well-supported clade (bootstrap value of 100%). Similarly, all the filamentous heterocystous cyanobacteria (including *Sphaerospermopsis* sp. LEGE 00249 and *Cuspidothrix* sp. LEGE 03284) cluster in a same clade. The three *Phormidium* LEGE strains have formed a tight subclade within a clade with other oscillatorialean species.

## 3. Discussion

As a consequence of the strain selection criteria, nearly half of the isolates tested here correspond to cyanobacterial genera for which the presence of cyanobactin genes had not been studied or discovered. We were able to detect cyanobactin genes in approximately 13% of the tested strains (85), a smaller proportion than that previously reported by Leikoski *et al*. [12], which have found cyanobactin A-genes in 36% of a varied set of 132 cyanobacterial strains belonging to the major taxonomic divisions. Nevertheless, Shih and co-workers [1] found similar results to the ones obtained in this study, having detected cyanobactin genes in 19% of the available cyanobacterial genomes (24 out of 126).

The present phylogenetic analysis of the A-gene employed a much larger number of sequences than previous studies and benefited from both our screening effort and from the genome sequencing studies that have been completed recently (e.g., [1]). The A-gene phylogeny allows the clear identification of six clades, which should correspond to different cyanobactin chemotypes (Figure 1). Leikoski *et al*. [12] have studied the phylogenetic relationships among A-gene sequences from 25 cyanobacteria. The resulting tree revealed clustering into the clades represented here as Clades I, II and VI. Later, and with a larger number of cyanobactin gene sequences available, Donia and Schmidt [6] identified four A-protease phylotypes and linked them to the corresponding chemotypes. These formerly described phylotypes and chemotypes are congruent with our analysis and correspond to four out of the six clades reported here, namely Clades I, II, IV and VI (Genotypes II, III, IV and I, respectively, in the afore-mentioned study). The remaining Clades, III and V, constitute in this manner two new putative cyanobactin groups resulting from this study.

Considering the previously defined groups, Clade I comprises cyanobacterial strains producing anacyclamides and prenylagaramides. The A-gene from the cyanobactin *osc* gene cluster (from *Oscillatoria* sp. PCC 6506) is also part of this clade, however its cyanobactin product has not been reported to date. As pointed out by Donia and Schmidt [6], cyanobactin clusters bearing the A-gene from this clade lead to the production of cyclic peptides without heterocyclization or oxidation, which is consistent with the absence of the cyclodehydratase (D) gene and oxidase domain (in gene G) (as an illustration see Supplementary Figure S1). The exception stands for the *osc* gene cluster, since while it features a similar gene arrangement than other cyanobactin synthetase clusters from this clade, it also contains all the genes required for the production of oxidated heterocyles (a characteristic of the chemotypes found in Clade VI). Hence, the *osc* gene cluster appears to be a hybrid pathway between those with their A-genes present in Clades I and VI. In fact, Donia and Schmidt [6] predict that the cyanobactin(s) encoded in this pathway will present structural features from both phylotypes. Moreover, this gene cluster may be an ideal candidate to study cyanobactin evolution.

Trichamide- and piricyclamide-producing strains are encompassed in Clade II. The *tri* gene cluster, from *Trichodesmium erythraeum* IMS101, has previously been considered an individual and separate phylotype because it presents distinctive characteristics. It contains most of the genes that are present in the gene clusters from Clade VI, but with a very different organization. Furthermore, the oxidase domain is present in a separate open reading frame [6] (as an illustration see Supplementary Figure S1). Our results show the inclusion of A-genes from the *pir* cluster in this clade. This can be explained because the proteases (A and G) from the *pir*-bearing strains *Microcystis aeruginosa* PCC 7005 and NIES-843 are almost identical to those of the *tri* pathway [14]. The A-gene from the *thc* gene cluster (*Cyanothece* sp. PCC 7425), placed in Clade IV, constitutes a separate genotype since the organization of this pathway is currently unique [6] (as an illustration see Supplementary Figure S1). Cyanobacterial strains that produce arthrospiramide (*Arthrospira platensis* NIES-39), patellamide, lissoclinamide, ulithiacyclamide, patellin and trunkamide (*Prochloron*), tenuecyclamide (*Nostoc spongiaeforme* var. *tenue* str. Carmeli), microcyclamide (*Microcystis aeruginosa*) and aesturamides, (*Lyngbya* sp. PCC 8106) fit in Clade VI of the A-gene phylogeny. Cyanobactin gene clusters present in this clade lead to the production of oxidated heterocyclic compounds, with the exception of the *tru* gene cluster [6] (as an illustration see Supplementary Figure S1).

The novel clades arising from this study (III and V), although corresponding only to gene A, are expected to be part of cyanobactin biosynthetic pathways different from those currently known. Thus, the study of the *Phormidium* sp., *Sphaerospermopsis* sp. and *Rivularia* sp. LEGE strains bearing these novel A-genes, both at the genetic and chemical levels, is of great interest. This potential becomes more evident if we take into account that even cyanobacterial strains with A-genes belonging to the same clade (e.g., Clade I and VI) are able to produce different types of cyanobactins.

Other A-gene sequences obtained from this study grouped into previously described clades. *Cuspidothrix* sp. LEGE 03284 is present in a sub-clade (Clade I) that contains anacyclamide-producing strains. In a similar manner, A-genes from all the *Microcystis* strains from this study are found in the same sub-clade (Clade II), which also contains the *pir*-bearing *Microcystis* strains. It is thus expectable that the aforementioned strains from this study will produce anacyclamides and piricyclamides, respectively. The study of such eventually produced cyanobactins should be of interest, in particular as piricyclamides and anacyclamides are known to display a considerable chemical diversity [14,22].

The existence of cyanobactin genes has been previously reported in *Anabaena* and *Aphanizomenon* [12] strains. However, recent taxonomic revisions have established several novel genera arising from the two aforementioned taxa. For instance, the case of the new proposed genus *Dolichospermum* [23], which comprehends the strain 1tu33s10, previously assigned to *Anabaena planctonica* (Figure 2). In contrast, the presence of cyanobactin synthetase genes in the genera *Cuspidothrix* and *Sphaerospermopsis*, which have also been recently established from *Anabaena* and *Aphanizomenon* taxonomic revisions [21,24,25], is unprecedented to our knowledge (Figure 2). This is also the first report of cyanobactin genes in the genus *Phormidium*.

The phylogeny of gene A also reveals several cyanobacterial strains, apart from those selected herein, from which new cyanobactins may be discovered. As an example, the *Nodularia spumigena* and *Snowella litoralis* with A-genes present in Clade I, are not known to produce cyanobactins, nor are other members of these genera. One other good candidate is *Cyanothece* sp. PCC 7822, which is present in the phylogenetic tree in a separate, distant branch from the cyanothecamide producer *Cyanothece* sp. PCC 7425. *Oscillatoria* strains present throughout the phylogeny, and in particular the strain containing the A-gene “loner sequence”, *Oscillatoria* sp. PCC 10802, represent potential candidates in the discovery of novel cyanobactins, which have not been reported for any member of this genus. Even though we could not retrieve the 16S rRNA gene sequence for this strain, the work of Shih *et al*. [1] allows inferring that this strain would be placed, in our phylogeny (Figure 2), in the clade harboring *Oscillatoria* spp. and *Phormidium* sp. sequences.

The lack of congruence between the A-gene and 16S rRNA gene phylogenies (Figure 1 and Figure 2), highlights that the ability to produce cyanobactins has, most probably, been laterally transferred (via horizontal transfer of gene clusters) from one taxon to another, as earlier suggested by Leikoski *et al*. [12]. This becomes more evident from the comparison of the cyanobacterial strains that are clustered in Clade VI (Figure 1) to their diffuse distribution among the 16S rRNA gene phylogeny (Figure 2). One other good example concerns the *Microcystis* spp. strains that are grouped separately (Clades II and VI, Figure 1), on the A-gene phylogeny and clustered together in the 16S rRNA gene phylogeny (Figure 2). Still, and as expected from previous studies, the phylogenetic tree (Figure 2) features the well-known polyphyly of most of the cyanobacterial orders [26,27,28], and even genera (as examples, see [28,29,30]), recognized in traditional systematics. However, the also documented monophyly of the heterocystous cyanobacteria [31] and that of the “traditional” genus *Microcystis* [32] was verified. The closely related *Phormidium* LEGE strains included in this study are placed within a clade with *Oscillatoria* spp., which, in turn, and unlike what can be seen in the A-gene phylogeny (Figure 1), have clustered together. These observations suggest that this particular group (*i.e.*, 16S rRNA gene phylotype) of filamentous non-heterocystous cyanobacteria can/may possess the ability to produce a variable set of currently unknown cyanobactins.

## 4. Experimental Section

### 4.1. Cyanobacterial Strains

A selection of cyanobacterial strains (Table 2 and Supplementary Figure S1) from the LEGE Culture Collection (Laboratory of Ecotoxicology, Genomics and Evolution; CIIMAR, Porto, Portugal) was performed in order to cover the diversity in the collection and the different ecosystems from which the strains were isolated. We tried to include cyanobacterial genera that had been tested in former studies [12], as well as genera that had not been previously examined. These were screened for the presence of the *N*-terminal protease (A) gene, and belonged to the orders Chroococcales (33 strains), Pleurocapsales (5 strains), Oscillatoriales (29 strains) and Nostocales (18 strains), according to the traditional systems of classification [33,34,35,36]. The isolates were originally obtained from freshwater (37 strains), estuarine (two strains) and marine (46 strains) environments. Additionally, three ESSACC (Estela Sousa e Silva Algal Culture Collection) strains [37] from freshwater environments were also included (hereafter named as LMECYA strains). 

### 4.2. Degenerate Primers

PCR primers were developed with the aim of being “universal” both in terms of taxa and cyanobactin diversity. For that purpose, publicly available (*i.e.*, in GenBank) cyanobactin A-gene sequences from different cyanobacterial taxa were aligned using the Clustal W algorithm [38] in the MEGA 5.05 software package [39]. Conserved regions among the homologous genes were thus identified and evaluated for candidate priming sites. This allowed us to design the primer set CBT_AF (5′-TTVGGYTAYGAYTTYGG-3′) and CBT_AR (5′-AGACCARGAACGRACTTC-3′), which amplify a 804 bp region of gene A. This primer pair was tested using a DNA template from *Microcystis aeruginosa* LEGE 91351 (formerly known as IZANCYA 41), a strain that was previously shown to be positive for the presence of cyanobactin genes [14]. Still, the corresponding A-gene sequence was obtained, as described below, and had not been reported yet. The PCR protocol was optimized, by performing reactions under an annealing temperature gradient (48 °C–60 °C, optimal 52 °C).

**Table 2:** Cyanobacterial genera tested for the presence of cyanobactin *N*-terminal protease (A) gene.

Order	Genus	Habitat(s) ^a^	Number of tested strains	Number of positive strains
Chroococcales	*Cyanobium*	M	9	0
	*Gloeocapsa*	M	1	0
	*Microcystis*	F	15	5
	*Synechococcus*	M	5	0
	*Synechocystis*	F; M	3	0
Pleurocapsales	*Chroococcopsis*	M	2	0
	*Chroococcidiopsis*	M	2	0
	*Hyella*	M	1	0
Oscillatoriales	*Leptolyngbya*	F; M	9	0
	*Nodosilinea*	M	2	0
	*Oscillatoria*	F	3	0
	*Phormidium*	F; E	4	3
	*Planktothrix*	F	3	0
	*Plectonema*	M	1	0
	*Pseudanabaena*	M	4	0
	*Romeria*	M	1	0
	*Schizothrix*	M	1	0
	Unidentified Pseudanabaenaceae	M	1	0
Nostocales	*Anabaena*	F	3	0
	*Aphanizomenon*	F	4	0
	*Calothrix*	M	3	0
	*Cuspidothrix*	F	1	1
	*Cylindrospermopsis*	F	2	0
	*Nodularia*	E	1	0
	*Nostoc*	M	1	0
	*Rivularia*	M	1	1
	*Scytonema*	M	1	0
	*Sphaerospermopsis*	F	1	1

**^a^** M—marine; F—freshwater; E—estuarine.

### 4.3. DNA Extraction, PCR Analyses and Sequencing

Exponentially growing cultures of each strain were harvested (1.5–2 mL) and the biomass centrifuged for 2 min at 7000× *g*. Total genomic DNA was extracted from cyanobacterial pellets using the Purelink Genomic DNA Mini Kit (Invitrogen, Carlsbad, CA, USA) following the manufacturer’s procedure for Gram-negative bacteria.

Cyanobacteria-specific primers 27F and CYA781R [40,41] and the primers CBT_AF and CBT_AR were used to amplify 16S rRNA genes (small subunit ribosomal gene) and cyanobactin A-genes, respectively, from the strains. PCR reactions were prepared in a volume of 20 µL containing 2.5 mM MgCl_2_, 125 µM of each deoxynucleotide triphosphate, 10 µM of each of the primers, 0.5 U of GoTaq^®^ DNA polymerase (Promega, Fitchburg, WI, USA) and 5–10 ng of DNA. Thermal cycling was carried out using a MyCycler (Bio-Rad, Hercules, CA, USA). In order to amplify the 16S rRNA gene, PCR analysis was performed with an initial denaturation step at 94 °C for 4 min followed by 30 cycles of 94 °C for 45 s, annealing temperature at 50 °C for 45 s, 72 °C for 1.20 min and a final extension step at 72 °C for 8 min. For the CBT_AF/CBT_AR primer-pair, thermal cycling conditions were as follows: initial denaturation at 94 °C for 3 min followed by 35 cycles of 94 °C for 45 s, annealing temperature at 52 °C for 45 s, 72 °C for 50 s and a final extension step at 72 °C for 7 min.

PCR products were separated in a 1.5% agarose gel in 1× TAE Buffer, stained with ethidium bromide (Bio-Rad, Hercules, CA, USA) and photographed under UV transillumination. DNA fragments from PCR products were gel extracted and purified with Cut&Spin DNA Gel Extraction Columns (Grisp, Porto, Portugal), following the manufacturer’s procedure. The purified DNA was then cloned into the pGEM^®^-T Easy Vector (Promega, Fitchburg, WI, USA). The vector was used to transform chemically competent *Escherichia coli* ONE Shot^®^ TOP10 cells (Invitrogen, Carlsbad, CA, USA). Plasmid DNA was isolated from the transformed cells using the GenElute^TM^ Plasmid Miniprep kit (Sigma-Aldrich, Saint Louis, MO, USA) and sequenced at Macrogen (Seoul, Korea) using M13/pUC sequencing primers.

### 4.4. Nucleotide Sequence Accession Numbers

16S rRNA gene and cyanobactin A-gene sequences obtained in this study were deposited in GenBank under the accession numbers [KC989698-KC989707] and [KF008255-KF008266], respectively (see also Table 1).

### 4.5. Phylogenetic Analyses

Phylogenetic studies of A-genes and 16S rRNA genes from the different cyanobacterial strains were performed. The putative A-gene sequences obtained in this study were independently submitted as the queries in a BLASTn search in order to confirm their identity. Since portions of the A- and G-genes are homologous, some of the sequences corresponded to the G-gene and were discarded. Cyanobactin A-gene sequences previously reported for cyanobacteria were also retrieved from the same database, as well as their respective 16S rRNA gene sequences (whenever available). Shih *et al*. [1] predicted cyanobactin synthetase gene clusters in several cyanobacterial genome sequences but in some cases, their genes were not fully annotated. Hence, we sought to identify the position of A-genes in the genome of those cyanobacterial strains by using anti SMASH [42] searches. This allowed retrieving the corresponding nucleotide sequences, but also identifying cyanobactin A-genes, hitherto not described in the literature, in other cyanobacterial genomes. These sequences were also included in the phylogenetic analyses.

MEGA 5.05 was used to perform the phylogenetic analyses. Multiple sequence alignments were obtained using the MUSCLE algorithm [43]. To compute the phylogenetic trees of A-genes and 16S rRNA genes, Maximum-Likelihood (ML) [44] methodology was employed using the model of evolution that best fitted the dataset. For both analyses, the Kimura 2-parameter model with gamma-distributed rate variation, using five substitution rate categories, and a proportion of invariant sites (K2P+Γ+I) was then selected, and 1000 bootstrap replicates were generated.

## 5. Conclusions

Phylogenetic analysis of cyanobactin *N*-terminal protease (A) genes seems to be an effective strategy to screen for cyanobacterial strains that may produce novel cyanobactins. Using this approach, two novel putative cyanobactin groups (Clades III and V, Figure 1) emerged, providing an up-to-date picture of cyanobactin A-gene diversity. The findings from this study emphasize the importance of prospecting the still uncovered cyanobacterial “tree of life”. Pursuing this will certainly expand the number of compounds (and chemotypes) from this important group of secondary metabolites.

## Supplementary Files

Supplementary File 1 Supplementary Information (PDF, 116 KB)
