# Supplementary material for: N-Terminal Protease Gene Phylogeny Reveals the Potential for Novel Cyanobactin Diversity in Cyanobacteria
Source: Mar Drugs. 2013 Dec 9;11(12):4902–16. doi: 10.3390/md11124902 (PMC3877893; doi:10.3390/md11124902)
Supplement: Supplementary File 1 — Supplementary Information (PDF, 116 KB) [file marinedrugs-11-04902-s001.pdf]

Supplementary Information

Table S1. Cyanobacterial isolates tested for the presence of cyanobactin *N*-terminal protease (A) gene.

Order	Isolates [a]	Habitat [b]	Origin [c]
Chroococcales	*Cyanobium* sp. LEGE 06012	M	Beach, Foz do Arelho, Portugal
	Cyanobium sp. LEGE 06098	M	Beach, Martinhal, Portugal
	Cyanobium sp. LEGE 06127	M	Beach, Burgau, Portugal
	Cyanobium sp. LEGE 06134	M	Beach, Moledo, Portugal
	Cyanobium sp. LEGE 06137	M	Beach, Lavadores, Portugal
	Cyanobium sp. LEGE 06138	M	Beach, Aguda, Portugal
	Cyanobium sp. LEGE 06140	M	Beach, Aguda, Portugal
	Cyanobium sp. LEGE 07153	M	Beach, Martinhal, Portugal
	Cyanobium sp. LEGE 07183	M	Beach, Olhos de Água, Portugal
	cf. *Gloeocapsa* sp. LEGE 06192	M	Beach, Burgau, Portugal
	Microcystis aeruginosa LEGE 08355	F	Reservoir, Torrão dam, Portugal
	Microcystis aeruginosa LEGE 91095	F	Reservoir, Torrão dam, Portugal
	Microcystis aeruginosa LEGE 91338	F	Coastal lagoon, Barrinha de Mira, Portugal
	Microcystis aeruginosa LEGE 91344	F	Pond, Lagoa dos Teixoeiros, Portugal
	Microcystis aeruginosa LEGE 91347	F	Reservoir, Bemposta dam, Portugal
	Microcystis aeruginosa LEGE 91351	F	Pond, Lagoa das Braças, Portugal
	Microcystis aeruginosa LMECYA 1	F	Reservoir, Montargil dam, Portugal
	Microcystis sp. AM VIVO A	F	Tâmega river, Portugal
	Microcystis sp. IZANCYA 45	F	Pond, Lagoa da Vela, Portugal
	Microcystis sp. LEGE 00258	F	Marco de Canaveses, Portugal
	Microcystis sp. LEGE 03282	F	Reservoir, Montargil dam, Portugal
	Microcystis sp. LEGE 08328	F	Lake, Zumpango, Mexico
	Microcystis sp. LEGE 08329	F	Man-made channel, Cuemanco, Mexico
	Microcystis sp. LEGE 08330	F	Artificial lake, Chapultepec, Mexico
	Microcystis sp. LEGE 08331	F	Man-made channel, Cuemanco, Mexico
	Microcystis sp. LEGE 08332	F	Man-made channel, Cuemanco, Mexico
	Synechococcus nidulans LEGE 06156	M	Beach, Burgau, Portugal
	Synechococcus nidulans LEGE 07186	M	Beach, Martinhal, Portugal
	Synechococcus sp. LEGE 06006	M	Beach, São Pedro de Moel, Portugal
	Synechococcus sp. LEGE 06026	M	Beach, Empa, Portugal
	Synechococcus sp. LEGE 06139	M	Beach, Aguda, Portugal
	Synechocystis salina LEGE 07173	M	Beach, Olhos de Água, Portugal
	Synechocystis sp. LEGE 00037	M	Beach, Moledo, Portugal
	Synechocystis sp. LEGE 07211	F	WWTP, Febros river, Portugal
Pleurocapsales	*Chroococcidiopsis* sp. LEGE 06147	M	Beach, Moledo, Portugal
	Chroococcidiopsis sp. LEGE 06174	M	Beach, Aguda, Portugal
	Chroococcopsis sp. LEGE 07161	M	Beach, Martinhal, Portugal
	Chroococcopsis sp. LEGE 07187	M	Beach, Moledo, Portugal
	Hyella sp. LEGE 07179	M	Beach, Moledo, Portugal
Oscillatoriales	*Leptolyngbya mycoidea* LEGE 06108	M	Beach, Luz, Portugal
	Leptolyngbya mycoidea LEGE 06118	M	Beach, Luz, Portugal
	Leptolyngbya mycoidea LEGE 07157	M	Beach, Lavadores, Portugal
	Leptolyngbya saxicola LEGE 06133	M	Beach, Moledo, Portugal

	Leptolyngbya saxicola LEGE 07132	M	Beach, Luz, Portugal
	Leptolyngbya saxicola LEGE 07170	M	Beach, Olhos de Água, Portugal
	Leptolyngbya sp. LEGE 06009	M	Beach, Foz do Arelho, Portugal
	Leptolyngbya sp. LEGE 06188	M	Beach, Lavadores, Portugal
	Leptolyngbya sp. LEGE 06362	F	WWTP, Febros river, Portugal
	Nodosilinea nodulosa LEGE 06152	M	Beach, Lavadores, Portugal
	Nodosilinea sp. LEGE 06120	M	Beach, Luz, Portugal
	Oscillatoria limnetica LEGE 00237	F	WTP, Mortágua, Portugal
	Oscillatoria sp. LEGE 03272	F	Coastal lagoon, Lagoa dos Salgados, Portugal
	Oscillatoria sp. LEGE 05292	F	Freshwater aquarium
	Phormidium sp. LEGE 06078	E	Douro estuary, Portugal
	Phormidium sp. LEGE 06204	F	WWTP, Febros river, Portugal
	Phormidium sp. LEGE 06363	F	WWTP, Febros river, Portugal
	Phormidium sp. LEGE 07215	F	WWTP, Febros river, Portugal
	Planktothrix sp. LEGE 07227	F	WWTP, Febros river, Portugal
	Planktothrix sp. LEGE 08334	F	Lake, Zumpango, Mexico
	Planktothrix sp. LEGE 08335	F	Lake, Zumpango, Mexico
	Plectonema cf. *radiosum* LEGE 06114	M	Beach, Luz, Portugal
	Pseudanabaena cf. *curta* LEGE 07169	M	Beach, Aguda, Portugal
	Pseudanabaena cf. *frigida* LEGE 06119	M	Beach, Burgau, Portugal
	Pseudanabaena cf. *frigida* LEGE 06144	M	Beach, Burgau, Portugal
	Pseudanabaena sp. LEGE 06116	M	Beach, Martinhal, Portugal
	Romeria sp. LEGE 06013	M	Beach, Arelho, Portugal
	Schizothrix aff. *septentrionalis* LEGE 07164	M	Beach, Moledo, Portugal
	Unidentified Pseudanabaenaceae LEGE 06148	M	Beach, Moledo, Portugal
Nostocales	*Anabaena* cf. *cylindrica* LEGE 00235	F	Reservoir, Maranhão dam, Portugal
	Anabaena sp. LEGE 00240	F	Reservoir, Maranhão dam, Portugal
	Anabaena sp. LEGE 04288	F	Marco de Canaveses, Portugal
	Aphanizomenon gracile LMECYA 40	F	Crato, Portugal
	Aphanizomenon (=*Cuspidothrix*) *issatchenkoi* LMECYA 31	F	Reservoir, Montargil dam, Portugal
	Aphanizomenon sp. LEGE 00251	F	Tábua, Portugal
	Aphanizomenon sp. LEGE 00238	F	Reservoir, Maranhão dam, Portugal
	Calothrix sp. LEGE 06100	M	Beach, Lavadores, Portugal
	Calothrix sp. LEGE 06122	M	Beach, S. Bartolomeu do Mar, Portugal
	Calothrix sp. LEGE 07177	M	Beach, Martinhal, Portugal
	Cuspidothrix sp. LEGE 03284	F	Montargil, Portugal
	Cylindrospermopsis raciborskii LEGE 97047	F	Queensland, Australia
	Cylindrospermopsis raciborskii LEGE 99045	F	Reservoir, Portugal
	Nodularia sp. LEGE 06071	E	Vouga estuary, Portugal
	Nostoc sp. LEGE 06150	M	Beach, S. Bartolomeu do Mar, Portugal
	Rivularia sp. LEGE 07159	M	Beach, Burgau, Portugal
	Scytonema sp. LEGE 07182	M	Beach, Moledo, Portugal
	Sphaerospermopsis sp. LEGE 00249	F	Reservoir, Maranhão dam, Portugal

[a] Strain in bold refers to positive control used in PCR reactions; strains underlined refer to lost cultures (*i.e.*, gDNA from lyophilized biomass); [b] M—marine, F—freshwater, E—estuarine; [c] WWTP—Wastewater Treatment Plant, WTP—Water Treatment Plant.

Figure S1. Schematic diagrams of representative cyanobactin gene clusters. (1) Example of the arrangement of a canonical cyanobactin gene cluster, *pat*, featuring the set of genes present in these pathways. A and G genes, coding for two proteases; E-gene, precursor peptide; B and C genes, coding for short conserved hypothetical proteins of unknown function. D and F genes, as well as an oxidase domain (in G-gene) lead, respectively, to heterocyclization, prenylation and oxidation of the resulting peptides; (2) Anacyclamide gene cluster, representing the genetic organization of cyanobactin pathways, lacking heterocyclases and oxidases; (3) Trichamide gene cluster, enlightening the presence of the oxidase domain in a separate open reading frame; (4) Cyanothecamide gene cluster, revealing the existence of more than one precursor peptide gene. (Adapted from [1]).

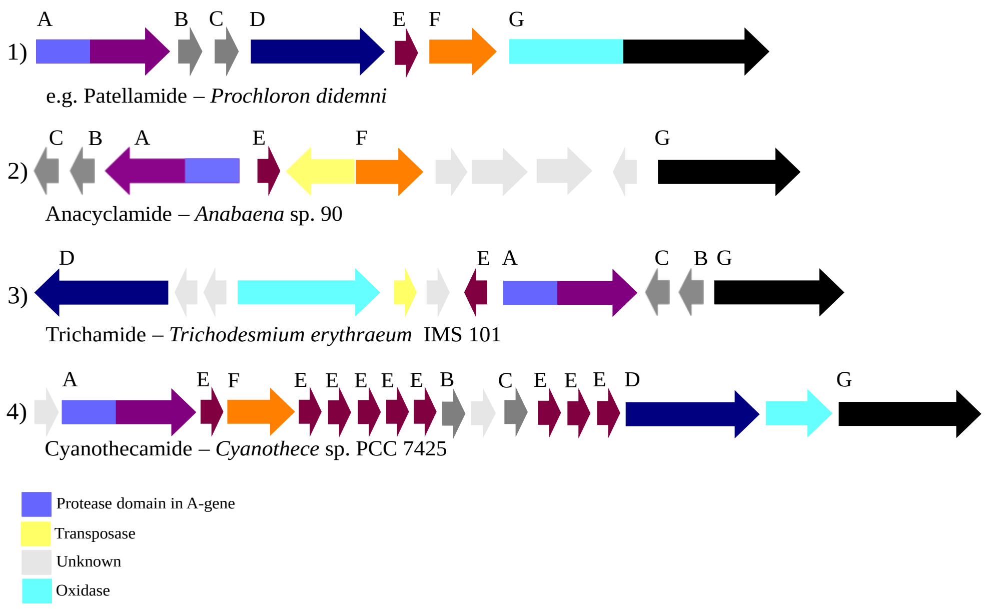

References

1. Donia, M.S.; Schmidt, E.W. Linking chemistry and genetics in the growing cyanobactin natural products family. *Chem. Biol.* **2011**, *18*, 508–519.

© 2013 by the authors; licensee MDPI, Basel, Switzerland. This article is an open access article distributed under the terms and conditions of the Creative Commons Attribution license (http://creativecommons.org/licenses/by/3.0/).
